# MiR-20a-5p Targeting the *TGFBR2* Gene Regulates Inflammatory Response of Chicken Macrophages Infected with Avian Pathogenic *E. coli*

**DOI:** 10.3390/ani14152277

**Published:** 2024-08-05

**Authors:** Xinqi Cao, Jiayi Ge, Yuyi Ma, Huan Li, Wei Han, Susan J Lamont, Hongyan Sun

**Affiliations:** 1 College of Animal Science and Technology, Yangzhou University, Yangzhou 225009, China; mx120230877@stu.yzu.edu.cn (X.C.); chencai2392@163.com (J.G.); mx120220887@stu.yzu.edu.cn (Y.M.); 2 School of Biological and Chemical Engineering, Yangzhou Polytechnic College, Yangzhou 225009, China; huan.li@me.com; 3 Jiangsu Institute of Poultry Sciences, Yangzhou 225003, China; sqbreedingg@126.com; 4 Department of Animal Science, Iowa State University, Ames, IA 50011, USA; nolanlisa@163.com

**Keywords:** gga-miR-20a-5p, APEC, *TGFBR2*, chicken macrophages, inflammatory response

## Abstract

**Simple Summary:**

Avian pathogenic *E. coli* (APEC) causes localized and systemic infections that are collectively known as avian colibacillosis. In the poultry industry, APEC infections lead to economic losses in the hundreds of millions of dollars per year and are a threat to human health. Given the diversity of APEC serotypes, it is important to understand the pathogenic mechanisms from a genetic perspective to control the disease. Increasing evidence indicates that microRNAs (miRNAs) are involved in host-pathogen interactions and immune responses. Our previous RNA-seq studies identified gga-miR-20a-5p as a key miRNA involved in the immune response of chicken macrophages to APEC infection. However, the related regulatory mechanism remains unclear. Here, we aimed to elucidate the role of gga-miR-20a-5p in the host defense against APEC in chickens and explore the underlying mechanisms. We found that gga-miR-20a-5p directly targeted transforming growth factor-beta receptor 2 (*TGFBR2*) by regulating its mRNA and protein expression and impacted the expression levels of pro-inflammatory cytokines, such as *IL8*, *TNFα*, *IL6*, and *IL1β*. The results of this study shed light on the role of gga-miR-20a-5p in the host defense against APEC.

**Abstract:**

Avian pathogenic *E. coli* (APEC) causes localized and systemic infections and are a threat to human health. microRNAs (miRNAs) play critical roles in inflammation and immune regulation following pathogen invasion. However, the related regulatory mechanism remains unclear. This study aimed to elucidate the involvement of chicken microRNA-20a-5p (gga-miR-20a-5p) in host defense against APEC in chickens and the underlying mechanisms. We evaluated the expression levels of gga-miR-20a-5p in chicken tissues and cells and observed a significant decrease in expression following APEC infection. Dual luciferase reporter assays showed that gga-miR-20a-5p directly targeted transforming growth factor-beta receptor 2 (TGFBR2), specifically by binding to the 3′-untranslated region (3′UTR) of *TGFBR2*. Overexpression of gga-miR-20a-5p markedly reduced both the mRNA and protein levels of TGFBR2, whereas inhibition of gga-miR-20a-5p significantly increased expression. Mechanistic investigations revealed that overexpression of gga-miR-20a-5p also attenuated the expression levels of the pro-inflammatory cytokines *IL8*, *TNFα*, *IL6*, and *IL1β*, whereas inhibition of gga-miR-20a-5p had the opposite effects. Collectively, our findings suggest that gga-miR-20a-5p regulates the immune response during APEC infection by targeting *TGFBR2*, thereby suppressing inflammatory cytokine production. This study provides valuable insights into the role of gga-miR-20a-5p in the host defense against APEC.

## 1. Introduction

Avian pathogenic *E. coli* (APEC) is a pathogenic bacterium that infects poultry, causing localized and systemic infections. APEC infection leads to a group of extraintestinal diseases collectively known as avian colibacillosis, including alveolitis, pericarditis, and peritonitis. The clinical manifestations of colibacillosis vary depending on age, health status, and the virulence factors of the infecting APEC strain [1]. Avian colibacillosis causes significant economic losses in the poultry industry due to decreased body weight, mortality, and carcass condemnation [2,3].

Current measures for preventing and treating APEC include vaccines and antibiotics [4,5]. However, increased antibiotic resistance among APEC strains and the limited effectiveness of vaccines against homologous strains have created an urgent demand for alternative methods to prevent and treat APEC infections. The utilization of host genetics to enhance disease resistance has emerged as a promising approach to combat the challenges posed by avian colibacillosis in poultry production [6]. Therefore, gaining a broader understanding of the factors that contribute to host resistance and susceptibility, and developing strategies to improve the disease resistance of the host, are a novel strategy for controlling avian colibacillosis.

MicroRNAs (miRNAs) are small non-coding RNA molecules consisting of 18–25 nucleotides. These RNAs regulate gene expression at the post-transcriptional level by marking target mRNAs for cleavage or inhibiting translation [7,8]. Previous RNA-seq studies identified gga-miR-20a-5p as a potential key miRNA involved in the immune response of chicken macrophages to APEC infection [9,10]. MiR-20a-5p has been investigated in various species, including humans (*Homo sapiens*) [11], mice (*Mus musculus*) [12], and chickens (*Gallus gallus*) [13]. For example, Zhang et al. showed that downregulation of miR-20a-5p induces apoptosis of human macrophages, contributing to host defense against mycobacterial infection [14]. Tian et al. showed that gga-miR-20a-5p mediates avian influenza virus (AIV)-induced immunosuppression in chickens by targeting the *NR4A3* gene [15]. Collectively, these studies suggest that miR-20a-5p is involved in vertebrate immune responses, which is consistent with our previous findings. However, the specific mechanism by which gga-miR-20a-5p influences the immune response of chicken macrophages to APEC infection remains unclear.

Transforming growth factor-beta receptor 2 (TGFBR2) is a transmembrane receptor that plays a crucial role in immune responses and various cellular functions, including cell proliferation, differentiation, and extracellular matrix production [16]. TGFBR2 forms a heterodimeric complex with TGF-beta receptor type-1 and binds to TGF-beta, and participates in both immunosuppressive and pro-inflammatory immune reactions [17]. Previous mRNA sequencing analyses showed a significant increase in the expression of chicken *TGFBR2* during APEC infection [18], and a bioinformatics analysis suggested a potential relationship between *TGFBR2* and gga-miR-20a-5p [19]. However, additional experimental verification was needed to confirm whether gga-miR-20a-5p regulates *TGFBR2*. This prompted further investigations into the roles of gga-miR-20a-5p in APEC infection and the potential interaction between *TGFBR2* and gga-miR-20a-5p.

This study aimed to investigate the role of gga-miR-20a-5p in the immune response of chicken macrophages during APEC infection and to elucidate the potential relationship between miR-20a-5p and *TGFBR2*.

## 2. Materials and Methods

### 2.1. Ethical Statement

The animal care procedures were conducted in strict compliance with the guidelines of the U.S. National Institute of Health (NIH Pub. No. 85–23, revised 1996). The experiments were approved by the Ethics Committee of Yangzhou University for Laboratory and Experimental Animals (Permit Number: YZUDWSY, Government of Jiangsu Province, China).

### 2.2. Tissues Collection

To investigate the expression levels of gga-miR-20a-5p and *TGFBR2* in chicken immune tissues following APEC infection, the spleen, blood, thymus, and bone marrow were collected from infected chickens as described in a previous study [20]. Briefly, 4-week-old male broiler chickens were challenged with APEC via the intra-air sac route, and another group of chickens was injected with PBS as a control. The tissues were collected from individuals with severe lesions at 5 days post infection.

To evaluate the expression patterns of gga-miR-20a-5p and *TGFBR2* in different chicken tissues, 1-day-old Rugao yellow chicks with uniform body weights were obtained from the Poultry Research Institute of the Chinese Academy of Agricultural Sciences (Yangzhou, China). The chicks were raised under standard conditions without vaccination. Eight 1-day-old chicks were euthanized via CO_2_ inhalation, and then thirteen tissue samples were harvested, including the cerebrum, heart, liver, spleen, lungs, stomach, cecum, bursa, small intestine, cerebellum, Harderian gland, muscles, and thymus. All collected tissues were immediately immersed in RNA preservation solution and frozen at −80 °C until RNA extraction.

### 2.3. Cell Culture and Passage

HD11 macrophages, an immortalized cell line of chicken myelocytomatosis type MC29 virus-transformed chicken hematopoietic cells, were obtained from ATCC (Manassas, VA, USA). The cells were cultured in DMEM supplemented with 10% fetal bovine serum (FBS) and maintained in a humidified chamber at 37 °C with 5% CO_2_. When the cells reached 80–90% confluence, they were passaged as described below. The culture medium was discarded, and the cells were washed twice with PBS. Then, the cells were treated with 1 mL of trypsin digestion solution for 3 min. Next, 2 mL of DMEM was added, and the mixture was centrifuged at 1000 rpm for 5 min. The supernatant was discarded, and fresh DMEM was added and mixed thoroughly. For all assays, HD11 cells between passages 20 and 30 were used.

### 2.4. RT-qPCR

Total RNA was isolated from cells and tissues using TRIzol reagent (Invitrogen, Carlsbad, CA, USA). Complementary DNA (cDNA) was subsequently synthesized using the One Step SYBR^®^ PrimeScript^®^ PLUS RT-PCR Kit (Takara, Dalian, China). To measure the expression levels of gga-miR-20a-5p, *TGFBR2*, and selected pro-inflammatory cytokines, RT-qPCR was performed using the SYBR^®^ Premix Ex Taq^TM^ II Kit (Takara, Dalian, China). The RT-qPCR cycling program consisted of an initial denaturation step at 95 °C for 3 min, followed by 40 cycles of denaturation at 95 °C for 10 s, annealing at 58 °C for 30 s, and extension at 72 °C for 30 s. U6 and *β-actin* were selected as the internal controls for miRNAs and genes, respectively. The primer sequences are listed in Table 1. The relative expression level of each gene or miRNA was calculated using the 2^−ΔΔCt^ method, where ΔΔCt was determined as follows: (Ct of gene/miRNA in the test group − Ct of *β-actin*/U6 in the test group) − (Ct of gene/miRNA in the control group − Ct of *β-actin*/U6 in the control group).

### 2.5. Bioinformatics Analysis of TGFBR2

ProtParam (http://web.expasy.org/protparam/; accessed on 6 May 2024) [21] was used to predict the molecular formula, molecular weight, isoelectric point (pI), and instability coefficient of TGFBR2. SignalP 4.1 (http://www.cbs.dtu.dk/services/SignalP-4.1/; accessed on 6 May 2024) [22] was performed to analyze the signal peptide of TGFBR2. TMHMM 2.0 (http://www.cbs.dtu.dk/services/TMHMM/; accessed on 6 May 2024) [23] was utilized to evaluate TGFBR2 for localization signals, secretory peptides, and transmembrane regions. The NetPhos 3.1 Server (http://www.cbs.dtu.dk/services/NetPhos/; accessed on 6 May 2024) [24] was used to predict potential threonine, serine, and tyrosine phosphorylation sites. Potential O- and N-glycosylation sites were predicted using the O-glycosylation sites (https://services.healthtech.dtu.dk/services/NetOGlyc-4.0/; accessed on 6 May 2024) [25] and NetNGlyc 3.1 Server (http://www.cbs.dtu.dk/services/NetNGlyc/; accessed on 6 May 2024), respectively. DNAMAN software (https://www.lynnon.com/dnaman.html; accessed on 6 May 2024) was used to construct a phylogenetic tree using the neighbor-joining method. The secondary structure, conserved domains, and three-dimensional homology of TGFBR2 were predicted using SOPMA and SWISS-MODEL software [26] (https://swissmodel.expasy.org/interactive; accessed on 6 May 2024). Finally, the protein–protein interaction (PPI) network of TGFBR2 was analyzed using the STRING database.

### 2.6. Prediction of Target Genes

The potential target genes of miR-20a-5p were analyzed by miRDB [27] and TargetScan [28].

### 2.7. Construction of Expression Vectors and Transient Transfection

Two plasmids, one containing the 3′-untranslated region (3′UTR) of *TGFBR2* that was predicted to bind gga-miR-20a-5p, and a second containing a mutation in this binding site, were constructed by Genecreate (Wuhan, China). gga-miR-20a-5p mimics and inhibitors were synthesized by GenePharma (Shanghai, China). For transient transfection, 1 × 10^5^ HD11 macrophages were seeded into each well of a 24-well plate. Then, 50 nM of a mimic/inhibitor/*TGFBR2* mutation vector, or the corresponding control, were transfected using Lipofectamine™ 8000 reagent (Invitrogen, Carlsbad, CA, USA) according to the manufacturer’s instructions. Following transfection, the cells were infected with 0.1 mL of 1 × 10^8^ CFU/mL of APEC O78 for 24 h. Control cells were treated with 0.1 mL of PBS for 24 h. After treatment, the cells were collected for subsequent experiments.

### 2.8. Dual-Luciferase Assay

The relationship between gga-miR-20a-5p and *TGFBR2* was investigated using a dual-luciferase assay. HD11 cells were transfected with 500 ng of either wild-type or mutant *TGFBR2* vector along with 500 ng of gga-miR-20a-5p mimic or inhibitor using Lipofectamine 8000™ transfection reagent (Invitrogen, Carlsbad, CA, USA) for 36 h. Then, luciferase activity was detected using the Dual-luciferase Reporter Assay Kit (Beyotime, Shanghai, China) according to the manufacturer’s instructions. The experiments were repeated independently four times, and each experiment was performed with triplicate samples.

### 2.9. Western Blotting

After the HD11 macrophages were incubated with mimic/inhibitor, with or without APEC infection, they were lysed with 200 μL of RIPA buffer (Beyotime Biotechnology, Shanghai, China). The lysed cells were centrifuged, and the supernatant was collected. The protein content was quantified using the BCA™ Protein Assay Kit (Pierce, Appleton, WI, USA). The isolated proteins were separated using sodium dodecyl sulfate-polyacrylamide gel electrophoresis (SDS-PAGE) and transferred onto PVDF membranes. The membranes were then blocked with 5% bovine serum albumin (BSA) for 2 h at room temperature (25 °C). The membranes were washed twice with PBST (PBS with 0.1% Tween-20) and then probed with the primary anti-TGFBR2 antibody (Invitrogen, Carlsbad, CA, USA) diluted 1:1000 at 4 °C overnight. Next, the membrane was incubated with the HRP-conjugated secondary antibody (Sigma-Aldrich, St. Louis, MO, USA) at room temperature (25 °C) for 2 h. Immunoblots were visualized using enhanced chemiluminescence (ECL kit; Santa Cruz Biotechnology, Dallas, TX, USA). The blots were visualized using Image Lab™ Software (Bio-Rad, Hercules, CA, USA).

### 2.10. Cell Viability Assay and Apoptosis Assay

Cell viability was assessed using the CCK-8 Kit (Solarbio, Beijing, China). Briefly, 1 × 10^5^ HD11 cells were seeded into 96-well culture plates. When the cells reached 70–80% confluence, they were transfected with gga-miR-20a-5p mimic or inhibitor for 6 h. Subsequently, the cells were infected with 0.1 mL of 1 × 10^8^ CFU/mL APEC O78 for 24 h. Then, the cells were incubated with 10 µL of CCK-8 solution for 2 h, and the absorbance was measured at 450 nm with a spectrophotometer. The experiment was repeated four times.

### 2.11. Nitric Oxide (NO) Production Assay

NO production in culture supernatants from the control, APEC-infected, gga-miR-20a-5p mimic-transfected combined with APEC-infected, and gga-miR-20a-5p inhibitor-transfected combined with APEC-infected HD11 cells were measured using Griess reagent kit (Molecular Probes, Carlsbad, CA, USA). The HD11 cell supernatant was mixed with Griess reagent and incubated in the dark for 30 min. Following incubation, the absorbance was measured at 540 nm with a spectrophotometer. The absorbance values were then compared to a sodium nitrite standard curve to determine the concentration of nitrite (μM) in each group.

### 2.12. Statistical Analysis

Data are expressed as the mean ± standard deviation (SD). For three or more groups, one-way analysis of variance (ANOVA) and Tukey’s honestly significant differences test (HSD; SAS, 2000; Cary, NC, USA) were conducted using JMP statistical software (version 15.2.1, SAS Institute). For two groups, the *t*-test was conducted using JMP statistical software. A *p* value less than 0.05 was considered statistically significant.

## 3. Results

### 3.1. APEC Infection Significantly Influenced the Expression of gga-miR-20a-5p and TGFBR2

**Figure 1:**
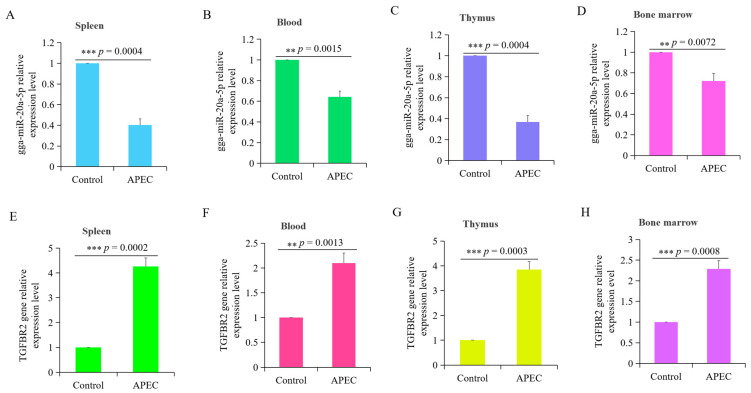
Expression patterns of gga-miR-20a-5p and *TGFBR2* gene were measured by RT-qPCR. (**A**–**D**) The expression levels of miR-20a-5p in the spleen (**A**), blood (**B**), thymus (**C**), and bone marrow (**D**) of chickens with APEC infection at day 5. (**E**–**H**) The relative expression levels of *TGFBR2* in the spleen (**E**), blood (**F**), thymus (**G**), and bone marrow (**H**) of chickens with APEC infection at day 5. Data represented as mean ± SD. n = 8; *t*-test; ns, not significant; ** *p* < 0.01; *** *p* < 0.001.

The expression levels of gga-miR-20a-5p and *TGFBR2* were assessed in four chicken tissues (spleen, blood, thymus, and bone marrow) following APEC infection. The results showed significant decreases in the expression of gga-miR-20a-5p in all four tissues (Figure 1A–D) following APEC infection. Conversely, the expression levels of *TGFBR2* in all four tissues were significantly increased following APEC infection (Figure 1E–H). Notably, the expression patterns of gga-miR-20a-5p and *TGFBR2* showed opposite trends during APEC infection.

### 3.2. Bioinformatics Analysis of gga-miR-20a-5p and TGFBR2

According to the information available on the NCBI and miRbase databases, in the chicken genome, miR-20a-5p is encoded on chromosome 1, spanning from 148,016,592 to 148,016,689 bp (Figure S1). Chicken miR-20a-5p consists of a single exon, and its mature sequence is UAAAGUGCUUAUAGUGCAGGUAG, with a length of 23 bases. The precursor sequence (pre-miR-20a-5p) is ugacagcucuuguagcacUAAAGUGCUUAUAGUGCAGGUAGuguucacuAAUCUACUGCAUUAUAAGCACUUAAAGUacugcuagcuguagaacuaca, with a length of 98 bp (Figure S2).

**Figure 2:**
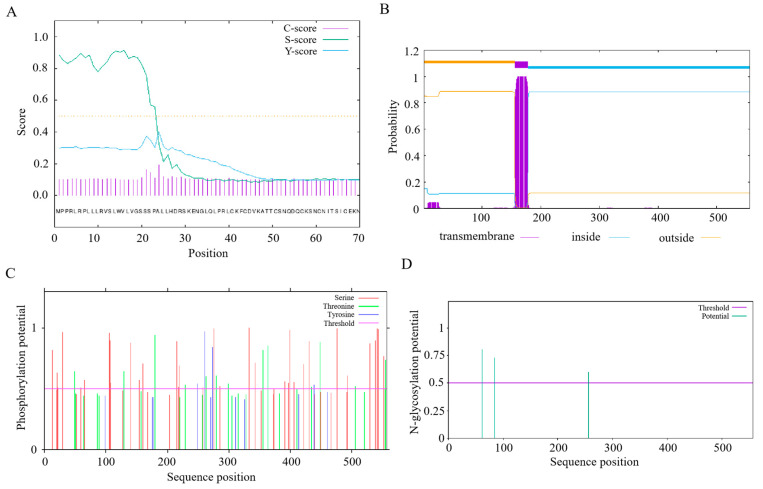
Bioinformatics analysis of TGFBR2. (**A**) Prediction of TGFBR2 signal peptide. (**B**) Prediction of TGFBR2 transmembrane domain. (**C**) Prediction of TGFBR2 phosphorylation sites. (**D**) Prediction of glycosylation sites in chicken TGFBR2.

The coding sequence (CDS) of *TGFBR2* is 1574 bp, which encodes a 557 amino acid protein. The protein has an aliphatic index of 82.75, and a grand average of hydropathicity (GRAVY) value of −0.447. Analysis using the peptide SignalP 4.1 server identified a putative signal sequence (Figure 2A), and an analysis using the TMHMM server indicated the presence of transmembrane domains in TGFBR2, with a total probability of the N-terminus being located in the cytoplasm of 0.14909 (Figure 2B). NetPhos predicted putative phosphorylation sites at 20 threonine, 47 serine, and 5 tyrosine residues in TGFBR2 (Figure 2C). NetOGlyc predicted one O-glycosylation site, and NetNGlyc identified potential N-glycosylation sites at residues 62, 84, and 257, with predicted rates of 0.8041, 0.7263, and 0.5997, respectively (Figure 2D). In summary, the TGFBR2 protein exhibits hydrophilic characteristics and harbors several putative phosphorylation sites.

**Figure 3:**
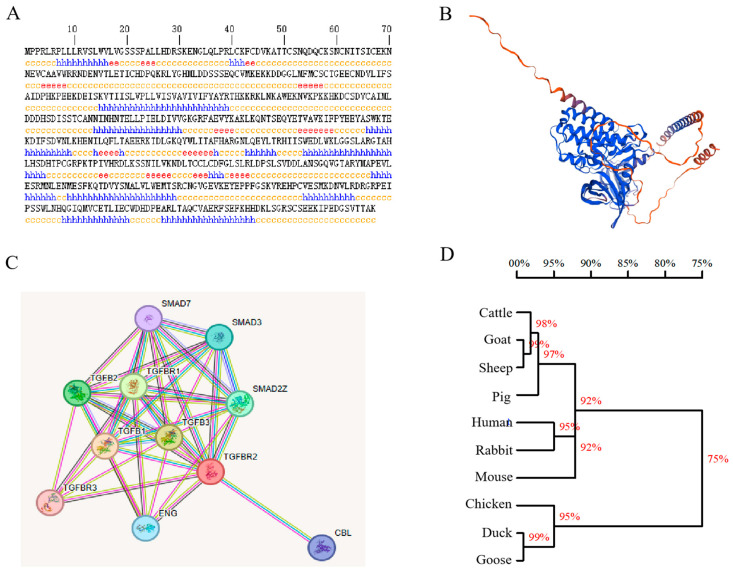
TGFBR2 protein structure, interaction network, and homologous strains among different species. (**A**) Prediction of TGFBR2 secondary structure. Note: h represents α-helix, e represents extended strand and c represents random coil. (**B**) Prediction of TGFBR2 tertiary structure. (**C**) TGFBR2 protein-protein interaction network. (**D**) TGFBR2 phylogenetic tree.

The secondary structure of TGFBR2 was predicted, which indicated that 59.43% of the amino acids form a random coil, 31.24% form an α-helix, and 9.34% form an extended strand (Figure 3A). The tertiary structure of TGFBR2 was predicted using SWISS-MODEL, which provided insights into its three-dimensional arrangement (Figure 3B). To explore possible interactions, a protein–protein interaction network for TGFBR2 was constructed using the STRING database, employing various criteria, such as co-expression, co-occurrence, text mining, experimental databases, neighborhood, and gene fusion. Through this analysis, 10 genes were identified as associated with TGFBR2 (Figure 3C). The evolutionary relationship of TGFBR2 protein among different animal species was analyzed using DNAMAN software. The resulting phylogenetic tree (Figure 3D) demonstrated that chickens and ducks/geese were clustered together, suggesting a high degree of homology among the three species.

### 3.3. Expression Pattern of gga-miR-20a-5p and TGFBR2

**Figure 4:**
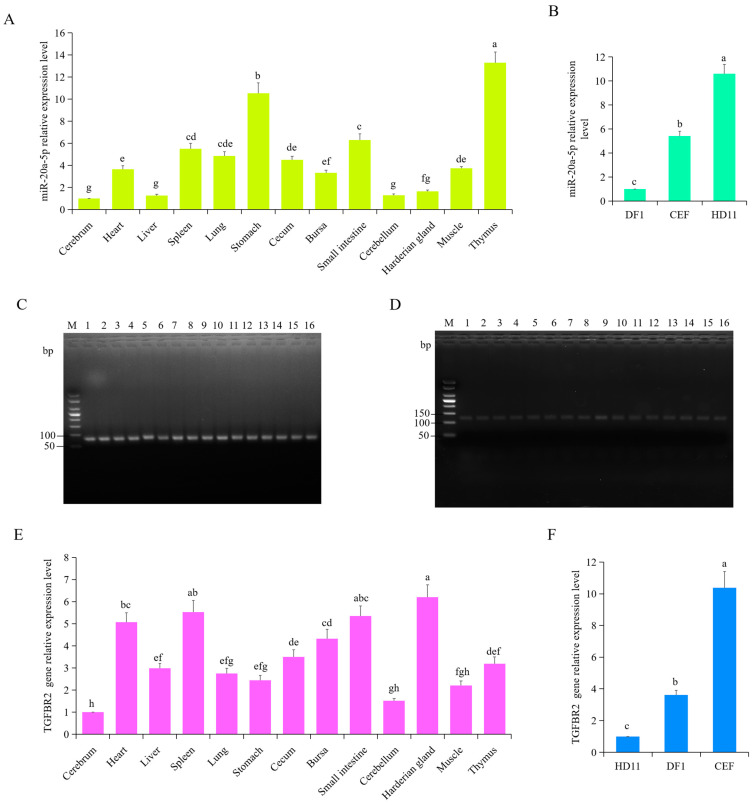
Expression patterns of gga-miR-20a-5p and *TGFBR2* in different chicken tissues and cells. (**A**,**E**). The relative expression levels of gga-miR-20a-5p (**A**) and *TGFBR2* (**E**) in cerebrum, heart, liver, spleen, lung, stomach, cecum, bursa, small intestine, cerebellum, Harderian gland, muscle, and thymus. β-actin gene was selected as an internal gene and cerebrum was chosen as the control. Data expressed as mean ± SD of eight individuals; ANOVA test; different letters indicate significant differences (*p* < 0.05); The same letters indicate insignificant differences (*p* > 0.05). (**B**,**F**) The relative expression levels of gga-miR-20a-5p (**B**) and *TGFBR2* (**F**) in HD11 macrophages, DF1 cells, and CEF cells. Data expressed as mean ± SD of 4 independent experiments; ANOVA test; different letters indicate significant differences (*p* < 0.05); The same letters indicate insignificant differences (*p* > 0.05). (**C**,**D**) RT-qPCR amplification products of gga-miR-20a-5p (**C**) and *TGFBR2* (**D**) were detected by agarose gel electrophoresis. Abbreviations: 1, cerebrum; 2, heart; 3, liver; 4, spleen; 5, lung; 6, stomach; 7, cecum; 8, bursa; 9, small intestine; 10, cerebellum; 11, Harderian gland; 12, muscle; 13, thymus; 14, HD11 macrophages; 15, DF1 cells; 16, CEF cells.

To investigate the tissue-specific expression patterns of chicken gga-miR-20a-5p and *TGFBR2*, total RNA was extracted from various tissues and cells, including the cerebrum, heart, liver, spleen, lung, stomach, cecum, bursa, small intestine, cerebellum, Harderian gland, muscle, and thymus, as well as DF1, CEF, and HD11 cells. RT-qPCR was performed to assess the relative expression levels of gga-miR-20a-5p and *TGFBR2* in these tissues. Expression of gga-miR-20a-5p was significantly higher in the thymus (*p* < 0.0001), stomach (*p* < 0.0001), small intestine (*p* < 0.0001), spleen (*p* < 0.0001), lung (*p* = 0.0011), cecum (*p* < 0.0001), muscle (*p* = 0.0005), and heart (*p* = 0.0011) than in the cerebrum tissue (Figure 4A). However, no significant differences in gga-miR-20a-5p expression were observed between the cerebrum and the Harderian gland, cerebellum, or liver (*p* > 0.05) (Figure 4A). The results also showed that HD11 cells had significantly higher expression levels of gga-miR-20a-5p than DF1 macrophages (*p* < 0.0001) and CEF cells (*p* < 0.0001) (Figure 4B).

Expression of *TGFBR2* was significantly higher in the Harderian gland (*p* < 0.0001), spleen (*p* < 0.0001), heart (*p* < 0.0001), bursa (*p* < 0.0001), cecum (*p* < 0.0001), thymus (*p* < 0.0001), liver (*p* = 0.0003), lung (*p* = 0.0025), and stomach (*p* = 0.0171) than in the cerebrum (Figure 4E). There was no significant difference in *TGFBR2* expression in the muscle and cerebellum when compared with that in the cerebrum (*p* > 0.05). The expression level of *TGFBR2* was higher in CEF cells than in DF1 (*p* < 0.0001) and HD11 cells (*p* < 0.0001) (Figure 4F). There were opposite expression patterns for *TGFBR2* and gga-miR-20a-5p in the Harderian gland, liver, and HD11 cells (Figure 4).

Agarose gel electrophoresis was used to verify the RT-qPCR products of gga-miR-20a-5p and *TGFBR2* in chicken tissues and cells, which confirmed amplification of the gga-miR-20a-5p and *TGFBR2* products (Figure 4C–D).

### 3.4. Time- and Dose-Dependent Effects of APEC Infection on the Expression Levels of gga-miR-20a-5p and TGFBR2

**Figure 5:**
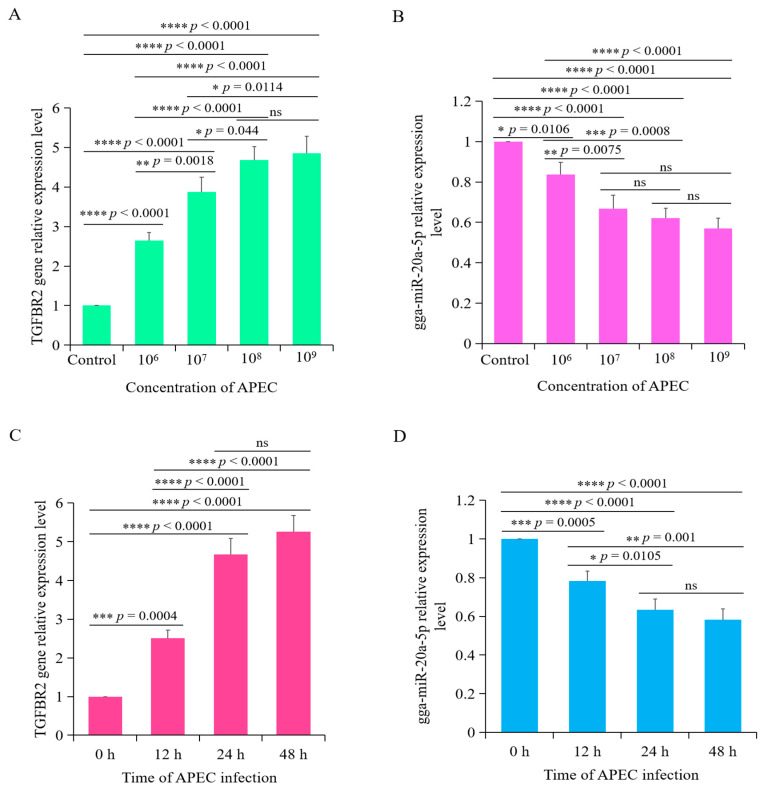
The correlation between gga-miR-20a-5p and *TGFBR2* during APEC infection. (**A**,**C**) gga-miR-20a-5p (**A**) and *TGFBR2* (**C**) expression in chicken macrophages with APEC infection at different dose (0, 10^6^ cfu/mL, 10^7^ cfu/mL, 10^8^ cfu/mL, and 10^9^ cfu/mL) for 24 h via RT-qPCR. Data expressed as mean ± SD of four independent experiments. ANOVA test; ns, not significant; * *p* < 0.05; ** *p* < 0.01; *** *p* < 0.001; **** *p* < 0.0001. (**B**,**D**) gga-miR-20a-5p (**B**) and *TGFBR2* (**D**) expression in chicken macrophages with APEC infection (1× 10^8^ cfu/mL) for 0 h, 12 h, 24 h, and 48 h via RT-qPCR. Data expressed as mean ± SD of four independent experiments. ANOVA test; ns, not significant; * *p* < 0.05; ** *p* < 0.01; *** *p* < 0.001; **** *p* < 0.0001.

To further investigate the correlation between gga-miR-20a-5p and *TGFBR2* during APEC infection, we analyzed their transcript levels in APEC-infected chicken macrophages at different time points and with different infectious doses. RT-qPCR showed that at 12 h after infection, *TGFBR2* expression was significantly higher in cells infected with 1 × 10^7^ cfu/mL APEC than in the uninfected control cells (Figure 5A). Expression of *TGFBR2* peaked at 1 × 10^9^ CFU/mL APEC, and there was no significant difference between *TGFBR2* expression levels at 1 × 10^8^ and 1 × 10^9^ CFU/mL APEC (Figure 5A). Expression of *TGFBR2* was rapidly induced upon infection with 1 × 10^8^ CFU/mL APEC and significantly increased over time, with the highest expression at 24 h post infection. Expression of *TGFBR2* was APEC dose- and infection time-dependent. In contrast, expression of gga-miR-20a-5p significantly decreased in response to increasing APEC dose (Figure 5C) and infection duration (Figure 5D). These findings revealed a negative correlation between gga-miR-20a-5p and *TGFBR2*.

### 3.5. TGFBR2 Was the Target Gene of gga-miR-20a-5p

**Figure 6:**
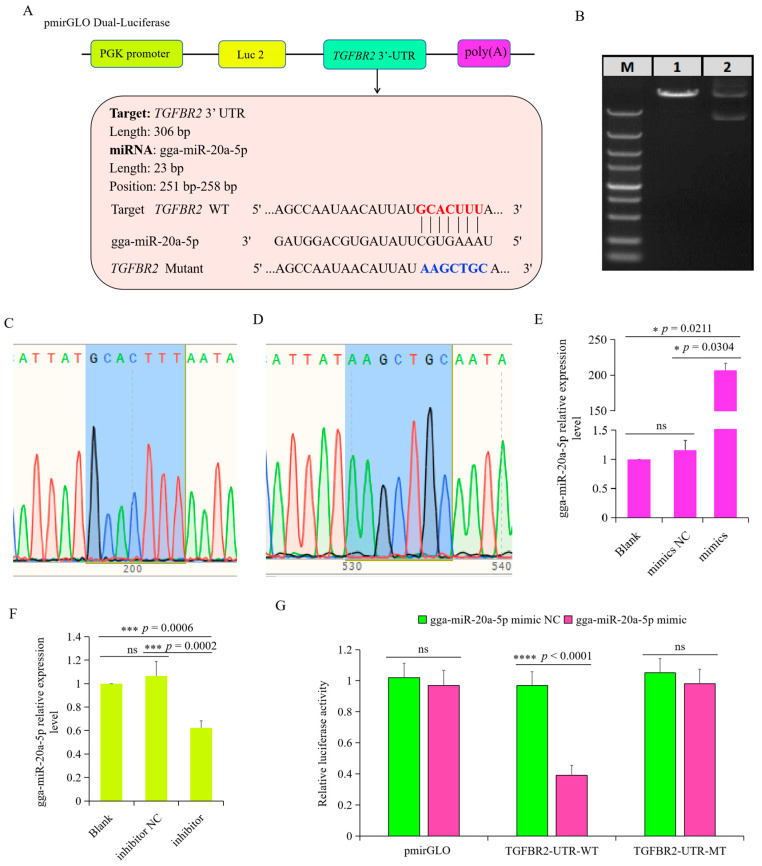
*TGFBR2* was the target gene of gga-miR-20a-5p. (**A**) The putative binding site for gga-miR-20a-5p and the 3′UTR of *TGFBR2*. (**B**) Double enzyme digestion of the pmirGLO plasmid for wild-type *TGFBR2*. M, marker; 1, plasmid digested by Sal I; 2, plasmid DNA. (**C**,**D**) The sequencing result of wild-type (**C**) and mutant (**D**) 3′ UTR of *TGFBR2*. (**E**,**F**) gga-miR-20a-5p expression in chicken macrophages transfected with gga-miR-20a-5p mimic (**E**) or inhibitor (**F**). Data expressed as mean ± SD of 4 independent experiments; ns, not significant; * *p* < 0.05; *** *p* < 0.001. (**G**). Luciferase activity was measured to identify relationship between gga-miR-20a-5p and *TGFBR2*. Data expressed as mean ± SD of n = 4 independent experiments; ns, not significant; **** *p* < 0.0001.

Preliminary predictions suggested *TGFBR2* as a potential target gene of gga-miR-20a-5p, and a putative binding site for gga-miR-20a-5p was identified in the 3′UTR of *TGFBR2* (Figure 6A). To directly test the relationship between gga-miR-20a-5p and *TGFBR2*, we constructed a luciferase reporter plasmid. The pmirGLO luciferase plasmid was digested with *Nhe I* and *Sal I* (Figure 6B) and ligated with *TGFBR2* fragments containing either a wild-type (WT) or mutant (MT) 3′UTR. Sequencing confirmed construction of the recombinant plasmids pmirGLO-TGFBR2-WT and pmirGLO-TGFBR2-MT (Figure 6C,D). RT-qPCR analysis of chicken macrophages transfected with either gga-miR-20a-5p mimic or inhibitor showed that the gga-miR-20a-5p mimic significantly increased the expression of gga-miR-20a-5p (Figure 6E), whereas the gga-miR-20a-5p inhibitor significantly decreased its expression (Figure 6F). To validate binding of gga-miR-20a-5p to the candidate target in the 3′UTR of *TGFBR2*, dual-luciferase reporter assays were performed in chicken macrophages after 36 h of co-transfection with pmirGLO-TGFBR2-WT or pmirGLO-TGFBR2-MT and either gga-miR-20a-5p mimic. The results showed that luciferase activity from the wild-type *TGFBR2* 3′UTR reporter plasmid was decreased by the gga-miR-20a-5p mimic (Figure 6G). However, this inhibition of luciferase activity by gga-miR-20a-5p was attenuated with the mutant *TGFBR2* 3′UTR construct. These findings provide evidence supporting the role of gga-miR-20a-5p as a direct regulator of *TGFBR2* in chickens.

### 3.6. TGFBR2 Was Regulated by gga-miR-20a-5p upon APEC Infection

**Figure 7:**
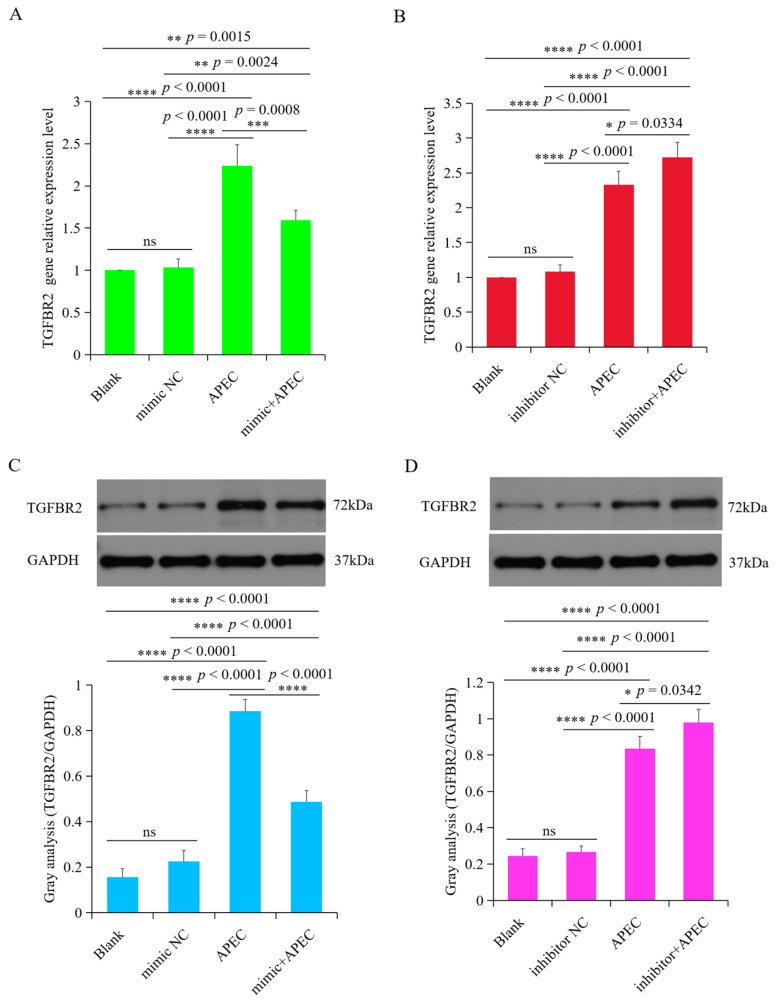
*TGFBR2* is regulated by gga-miR-20a-5p upon APEC infection. (**A**,**B**) The mRNA expression of *TGFBR2* in chicken macrophages transfected with gga-miR-20a-5p mimic (**A**) or inhibitor (**B**) upon APEC infection. Data expressed as mean ± SD of 4 independent experiments; ANOVA test; ns, not significant; * *p* < 0.05; ** *p* < 0.01; *** *p* < 0.001; **** *p* < 0.0001. (**C**,**D**). The protein expression level of *TGFBR2* in chicken macrophages transfected with gga-miR-20a-5p mimic (**C**) or inhibitor (**D**) upon APEC infection. Data expressed as mean ± SD of 4 independent experiments; ANOVA test; ns, not significant; * *p* < 0.05; **** *p* < 0.0001. The original Western blot figures can be found in Supplementary Materials.

To investigate the regulatory effects of gga-miR-20a-5p on *TGFBR2* during APEC infection, we examined the mRNA and protein expression levels of TGFBR2 in chicken macrophages treated with either a gga-miR-20a-5p mimic or inhibitor during APEC infection. The results showed that overexpression of gga-miR-20a-5p significantly suppressed the expression of TGFBR2 at both the mRNA and protein levels during APEC infection compared with the levels in the APEC infection group (Figure 7A,C). Conversely, inhibition of gga-miR-20a-5p increased expression of TGFBR2 at both the mRNA and protein levels during APEC infection (Figure 7B,D). These findings, in conjunction with the luciferase reporter assay results, provide comprehensive evidence that gga-miR-20a-5p directly targets the 3′UTR of *TGFBR2* and participates in its regulation during APEC infection.

### 3.7. gga-miR-20a-5p Regulated the Expression of Downstream Inflammatory Mediators of TGFBR2 during APEC Infection

**Figure 8:**
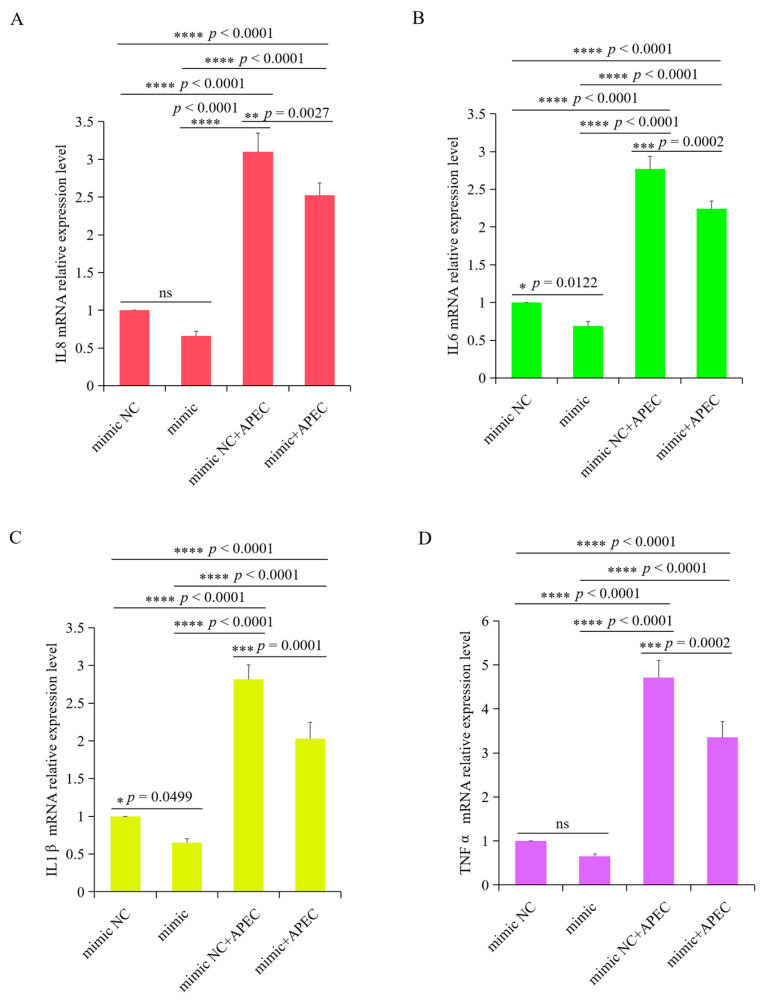
Overexpression of gga-miR-20a-5p significantly decreased the expression level of *IL8* (**A**), *IL6* (**B**), *IL1β* (**C**), and *TNFα* (**D**) upon APEC infection. Data expressed as the mean ± SD of 4 independent experiments; ANOVA test; ns, not significant; * *p* < 0.05; ** *p* < 0.01; *** *p* < 0.001; **** *p* < 0.0001.

**Figure 9:**
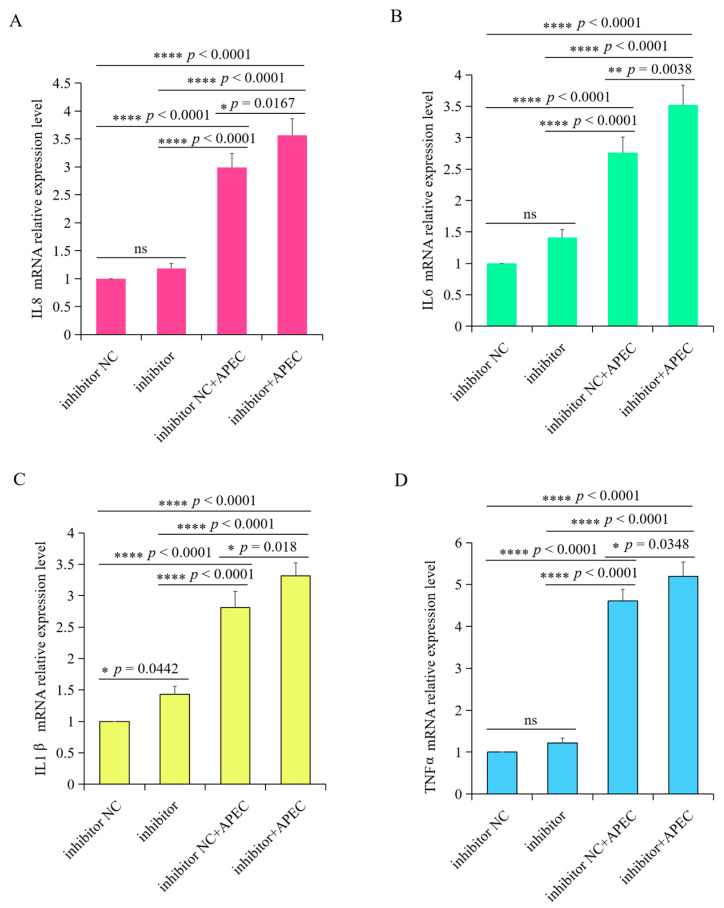
gga-miR-20a-5p inhibitor significantly increased the expression level of *IL8* (**A**), *IL6* (**B**), *IL1β* (**C**), and *TNFα* (**D**) upon APEC infection. Data expressed as mean ± SD of 4 independent experiments; ANOVA test; ns, not significant; * *p* < 0.05; ** *p* < 0.01; **** *p* < 0.0001.

To assess the impact of gga-miR-20a-5p on the inflammatory response triggered by APEC infection, we examined the expression levels of *IL8*, *IL1β*, *IL6*, and *TNFα* in chicken macrophages transfected with the gga-miR-20a-5p mimic or inhibitor for 36 h, both with and without APEC infection. APEC infection significantly upregulated the expression of these cytokines and induced the release of inflammatory mediators from chicken macrophages (Figure 8 and Figure 9). However, when the gga-miR-20a-5p mimic was introduced, there was a significant decrease in the expression levels of *IL8*, *IL1β*, *IL6*, and *TNFα* during APEC infection compared with the levels in APEC-infected cells (Figure 8). Conversely, in gga-miR-20a-5p inhibitor-transfected cells, the expression levels of these cytokines were significantly increased upon APEC infection compared to those in APEC infected cells without inhibitor (Figure 9). These results collectively indicated that gga-miR-20a-5p functions as an antibacterial factor in the cellular response to APEC infection by modulating the expression of key inflammatory cytokines.

### 3.8. gga-miR-20a-5p Influenced Cell Viability and NO Production upon APEC Infection

**Figure 10:**
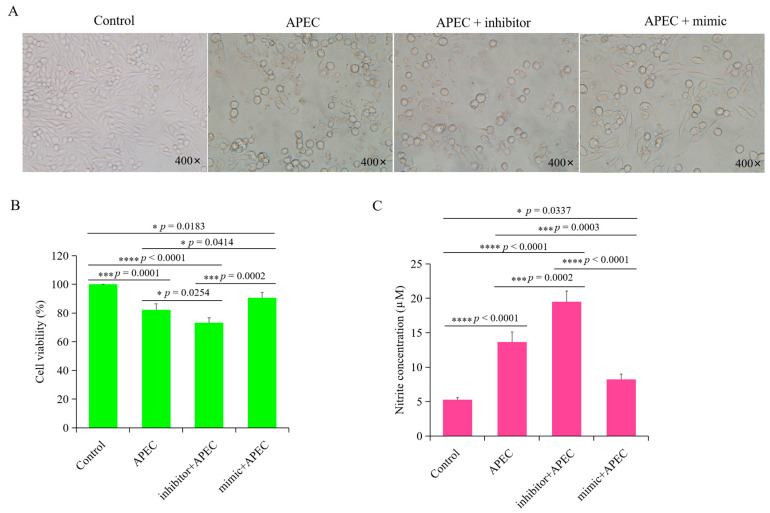
Effect of gga-miR-20a-5p on cell viability and NO production during APEC infection. (**A**) HD11 cells morphology in the groups of control, APEC infection, inhibition of gga-miR-20a-5p + APEC, and overexpression of gga-miR-20a-5p + APEC. (**B**) Cell viability of HD11 in the groups of control, APEC infection, inhibition of gga-miR-20a-5p + APEC, and overexpression of gga-miR-20a-5p + APEC. Data expressed as mean ± SD of 4 independent experiments; ANOVA test; ns, not significant; * *p* < 0.05; *** *p* < 0.001; **** *p* < 0.0001. (**C**). NO production in HD11 cells in the groups of cControl, APEC infection, inhibition of gga-miR-20a-5p + APEC, and overexpression of gga-miR-20a-5p + APEC. Data expressed as mean ± SD of 4 independent experiments; ANOVA test; ns, not significant; * *p* < 0.05; *** *p* < 0.001; **** *p* < 0.0001.

To further assess the effects of gga-miR-20a-5p during APEC infection, we examined cell viability and NO production in uninfected control cells, APEC-infected cells, gga-miR-20a-5p-overexpressing + APEC-infected cells, and gga-miR-20a-5p inhibition + APEC-infected cells. The results showed that both the APEC infection group and the gga-miR-20a-5p inhibition + APEC infection group exhibited noticeable cytopathic effects (Figure 10A). However, overexpression of gga-miR-20a-5p significantly mitigated the cytopathic effects induced by APEC infection (Figure 10A). Analysis of cell viability using the CCK8 assay showed significantly higher cell viability in gga-miR-20a-5p overexpression combined with APEC infection group than in both the APEC infection group (*p* = 0.0414) and gga-miR-20a-5p inhibition-transfected + APEC infection group (*p* = 0.0002) (Figure 10B).

Both APEC infection alone and gga-miR-20a-5p inhibition combined with APEC infection resulted in significant increases in NO production in chicken macrophages compared with the levels in the uninfected control macrophages (*p* < 0.0001 and *p* < 0.0001, respectively), and NO levels were higher in the gga-miR-20a-5p inhibition combined with APEC infection group than in the APEC infection group (*p* = 0.0002) (Figure 10C). However, NO production in the gga-miR-20a-5p overexpression + APEC infection group was significantly lower than that in both the APEC infection group (*p* = 0.0003) and the gga-miR-20a-5p inhibition + APEC infection group (*p* < 0.0001). Notably, a significant difference in NO production was observed between the control and gga-miR-20a-5p overexpression + APEC infection groups (*p* = 0.0337) (Figure 10C). These findings suggest that overexpression of gga-miR-20a-5p can effectively reduce NO production during APEC infection.

## 4. Discussion

Emerging evidence indicates that miRNAs play crucial roles in the regulation of innate and adaptive immune responses against various bacterial infections, particularly the Gram-negative pathogen APEC [29]. For example, let-7i-3p miRNA has been shown to potently inhibit replication of *Salmonella* by modulating endolysosomal trafficking and the vacuolar environment via targeting the host RGS2 protein [30]. Overexpression of gga-miR-429 significantly suppressed the expression of *TMEFF2* and *SHISA2*, thereby regulating the platelet-derived growth factor (PDGF) and Wnt signaling pathways following APEC infection in chicken HD11 macrophages [31]. Zhao et al. reported that miR-200c-3p attenuated lipopolysaccharide (LPS)-induced inflammatory responses by targeting *RIP2* [32]. Despite these valuable insights, our understanding of the specific functions of miRNAs in host responses to APEC infection in chickens remains limited. Therefore, further investigations are necessary to elucidate the roles of miRNAs in modulating inflammatory responses against APEC infection in chickens and the related mechanisms. These studies should advance our knowledge of pathogenesis, enhance animal welfare, reduce economic losses in the poultry industry, and ensure food safety.

To date, no published study has reported the specific regulation of immune responses by miR-20a-5p during APEC infection in chickens. In other words, the precise functions of miR-20a-5p in the modulation of innate immunity during APEC infection are unknown and warrant further investigation. Our preliminary transcriptome sequencing data revealed that the expression of gga-miR-20a-5p differed significantly between healthy and APEC-infected chickens [33]. Accordingly, we hypothesized that gga-miR-20a-5p is involved in regulating the host immune response to APEC infection. Moreover, the mechanism by which gga-miR-20a-5p regulates inflammatory responses in chickens may be a novel finding.

In the present study, we found that the expression of gga-miR-20a-5p was significantly downregulated in the spleen, blood, bone marrow, and thymus following APEC infection. Consistent with our findings, Su et al. reported a decrease in the expression of gga-miR-20a-5p in the spleen and bursa of Fabricius during avian influenza virus (AIV) infection [34]. Macrophages are the first line of defense against pathogens and when activated, they produce NO [35] and upregulate the expression of pro-inflammatory cytokines, such as IL1β, TNFα, and IL6 [36]. In the current investigation, we showed that overexpression of gga-miR-20a-5p significantly increased NO production and reduced the expression levels of the pro-inflammatory cytokines *IL1β*, *IL6*, *IL8*, and *TNFα* during APEC infection. Conversely, inhibition of gga-miR-20a-5p had the opposite effects. These findings indicate that gga-miR-20a-5p is involved in the regulation of host immune and inflammatory responses against APEC infection. This observation is consistent with the findings in a study by Hong et al. [13], in which overexpression of gga-miR-20a-5p significantly downregulated the expression of *IFNγ*, *IL1β*, and *TNFα* in response to poly(I:C) stimulation. Collectively, these results suggest that chicken gga-miR-20a-5p plays a similar role in modulating the immune response, regardless of whether the pathogenic challenge is bacterial or viral.

Importantly, owing to their post-transcriptional regulatory effects, the function of a given miRNA depends primarily on the regulation of its target gene(s) [37]. Consequently, identifying the target mRNA(s) of each miRNA is critical for a thorough understanding of its biological functions. Notably, we identified *TGFBR2* as a direct target of gga-miR-20a-5p and demonstrated that it is regulated by gga-miR-20a-5p during APEC infection. TGF-beta is a multifunctional cytokine with profound effects on the immune system that is considered one of the most potent immunosuppressive agents and plays a key role in promoting tumorigenesis [38,39,40]. TGFBR2 is one of the receptors for TGF-beta, and thus it plays an important role in TGF-beta-related pathways and signal transduction [41]. Cao et al. reported that TGFBR2 expression was significantly increased in a time-dependent manner following treatment with lipopolysaccharide (LPS) [42]. Consistent with this finding, we observed that chicken TGFBR2 expression was upregulated in a time- and infective dose-dependent manner following APEC infection.

Chicken TGFBR2 shares a moderate degree of homology to human TGFBR2 (75%). Yang et al. showed that knockdown of *TGFBR2* ameliorated LPS-induced inflammation and apoptosis in human kidney-2 (HK2) cells [43]. Taken together, our results and those of previous studies suggest that gga-miR-20a-5p modulates the inflammatory cytokine response by targeting *TGFBR2* during APEC infection.

## 5. Conclusions

The results of this study preliminarily confirm that downregulation of gga-miR-20a-5p in tissues infected with APEC promotes the release of pro-inflammatory cytokines by suppressing its target gene *TGFBR2*. These findings contribute to a deeper understanding of the pathogenesis of APEC in chickens. Our study provides new insights into the mechanisms underlying the host immune response to APEC infection through miRNA-mediated regulation and offers potential guidance for the identification of novel vaccine targets.

## Figures and Tables

**Table 1:** Primers for candidate genes/miRNAs.

Name	Accession Number	Forward (5′-3′)	Reverse (5′-3′)
*TGFBR2*	XM_046910484.1	TCTTGTCCCTTTATTGGTG	TTATGTTTCTTGGGCTTGA
*β-actin*	NM_205518.2	CAGCCAGCCATGGATGATGA	ACCAACCATCACACCCTGAT
*IL1β*	XM_046931582.1	GCCGAGGAGCAGGGACTTT	ACTGTGAGCGGGTGTAGCG
*IL8*	NM_205018.2	GAGTTCACTGACCACCCT	TGCCTGAGCCATACCTTT
*IL6*	NM_204628.2	TTATGGAGAAGACCGTGAG	GTGGCAGATTGGTAACAGA
*TNFα*	XM_046900549.1	CGTTCGGGAGTGGGCTTTA	TTGTGGGACAGGGTAGGG
*gga-miR-20a-5p*	NR_031405.1	TAAAGTGCTTATAGTGCAGGTAG	CAGTGCGTGTCGTGGAGT
*U6*	XM_025154275.3	CAAGGACCCATCGTTCCACA	CCATTGGACACGCAGAATGC

## Data Availability

The data will be available from the corresponding author upon request.

## Supplementary Materials

The following supporting information can be downloaded at: https://www.mdpi.com/article/10.3390/ani14152277/s1. Figure S1. The information of chicken miR-20a-5p from NCBI database; Figure S2. The precursor sequence (pre-miR-20a-5p) of chicken miR-20a-5p; File S1. Original western blot.
