# Supplementary material for: MiR-20a-5p Targeting the TGFBR2 Gene Regulates Inflammatory Response of Chicken Macrophages Infected with Avian Pathogenic E. coli
Source: Animals (Basel). 2024 Aug 5;14(15):2277. doi: 10.3390/ani14152277 (PMC11311048; doi:10.3390/ani14152277)
Supplement: Supplementary file 1 [file animals-14-02277-s001.zip › animals-3091350-File S1.pdf]

Original western blot

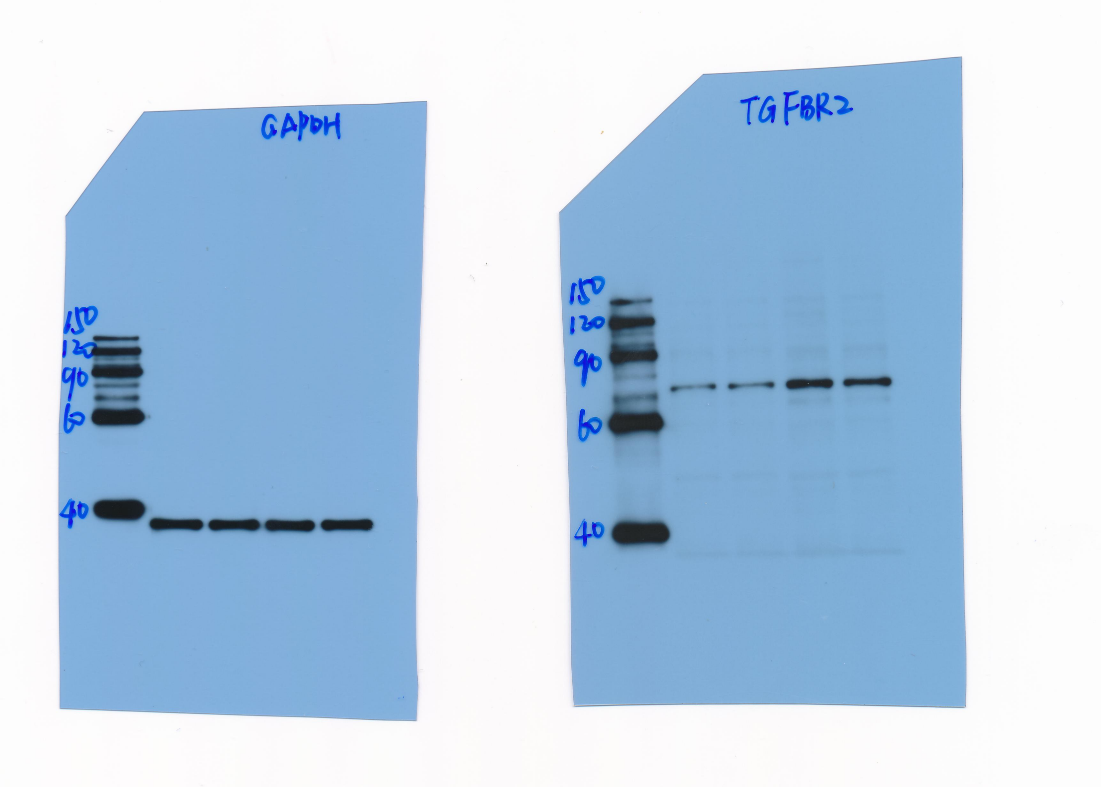

This is the original western blot of figure 7C.

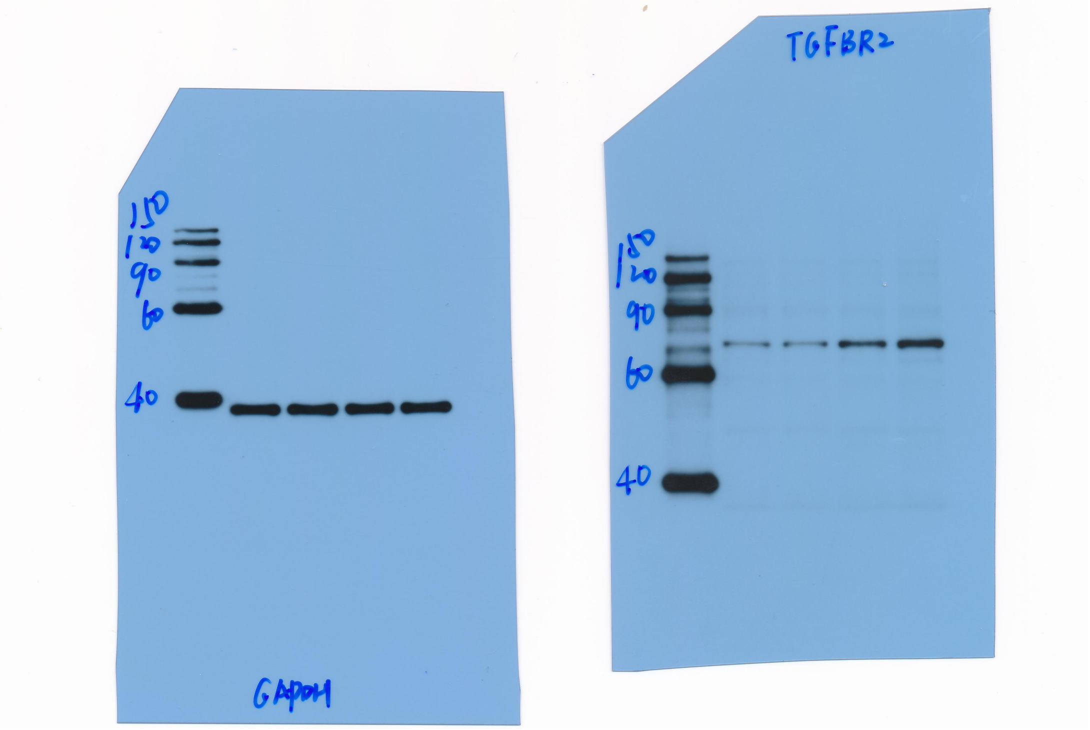

This is the original western blot of figure 7D
