# Supplementary figures and images for: MiR-20a-5p Targeting the TGFBR2 Gene Regulates Inflammatory Response of Chicken Macrophages Infected with Avian Pathogenic E. coli
Source: Animals (Basel). 2024 Aug 5;14(15):2277. doi: 10.3390/ani14152277 (PMC11311048; doi:10.3390/ani14152277)

Supplementary Materials

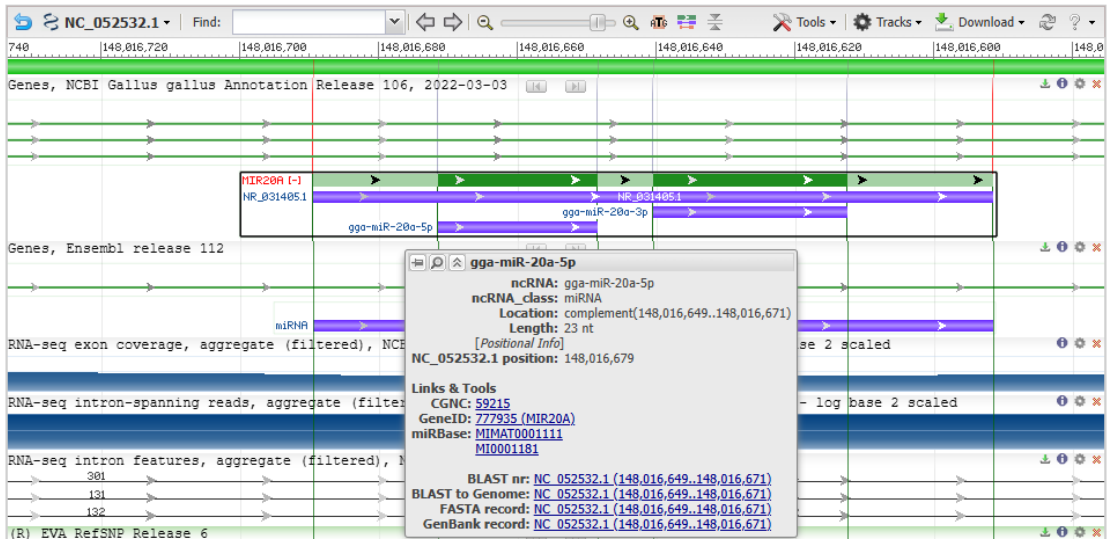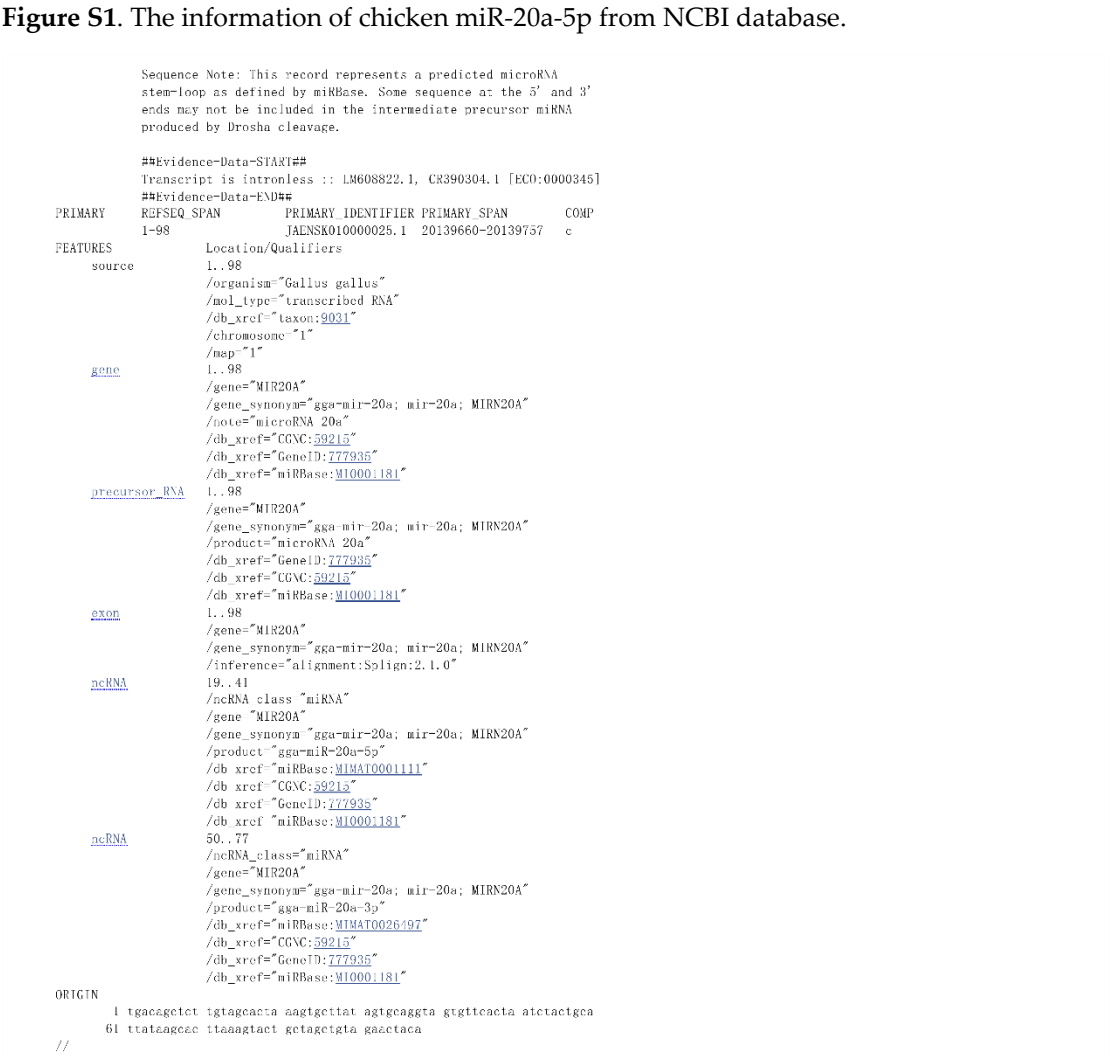

Figure S2. The precursor sequence (pre-miR-20a-5p) of chicken miR-20a-5p.

Supplement: Supplementary file 1 [file animals-14-02277-s001.zip › animals-3091350-Figures S1 and S2.pdf]
